# Anticipation (second-order motor planning) is stored in memory – processing of grasp postures in a priming paradigm

**DOI:** 10.3389/fpsyg.2024.1393254

**Published:** 2024-07-17

**Authors:** Jonas Kämpfer, Ludwig Vogel, Thomas Schack

**Affiliations:** ^1^Neurocognition and Action – Biomechanics Research Group, Faculty of Psychology and Sports Science, Bielefeld University, Bielefeld, Germany; ^2^Center for Cognitive Interaction Technology (CITEC), Bielefeld University, Bielefeld, Germany

**Keywords:** end-state comfort, grasping, action anticipation, action representation, priming, motor planning

## Abstract

The *end-state comfort effect* (ESC) describes the tendency to grasp an object with an initial uncomfortable grasp posture in order to achieve a comfortable end posture. The ESC is an example for anticipative processes in manual action. ESC planning is investigated in many studies where this effect is measured in the context of motor observation and motion capture. However, there is little evidence if the anticipative link between different action states, especially between initial grasp postures and comfortable end postures, is represented in memory. The aim of the present study was to investigate whether the perception of a grasp posture holding a bar leads to the activation of action-related representations of grasping actions. For this purpose, a priming paradigm was used in which prime images were shown depicting either a comfortable (overhand grip) or uncomfortable (underhand grip) grasp posture holding a two-colored bar. The subsequently shown target images represented either a comfortable (thumb-up) or uncomfortable (thumb-down) final grasp posture of this grasping action. Due to the different grasp postures in the prime and target, prime-target pairs represented different types of action sequences. Furthermore, physically possible, and physically impossible actions were presented. Participants were asked to react to the top color of the bar shown in the target-picture, whereby the shown grasp posture was irrelevant for this decision. Results showed that reaction times did not differ after presentation of an overhand grip to target pictures showing comfortable or uncomfortable final grasp postures. In contrast, after presentation of an underhand grip in the prime, reactions to target pictures with final comfortable grasp postures were faster compared to target pictures with uncomfortable grasp postures. The effect was only found for the physically possible action. The findings suggest that the perception of the underhand grip leads to cognitive pre-activation of a final action state. The present study suggests that the association between an initial uncomfortable underhand grip and its action effect, in form of a final action state that is consistent with the ESC, is represented in memory. Such motor representation might be important for the anticipation and control of goal-directed grasping.

## 1 Introduction

In a large part of everyday actions, we use our hands to interact with one or more objects. For example, when we want to drink, we need a glass, when we eat, we need cutlery, or when we write a text on a computer, we need a keyboard. Accordingly, we need our hands to interact with the environment and to be able to act in it. The way we grasp objects depends on various factors such as the perceived physical properties of the object, such as its size, orientation and distance (Castiello, 2005; Desanghere and Marotta, 2015; Ansuini et al., 2016), but furthermore the goal of the action (Armbrüster and Spijkers, 2006; Rosenbaum et al., 2006; Ansuini et al., 2008; Rosenbaum et al., 2012; Cienfuegos et al., 2022). The former, i.e., the planning of actions on the basis of immediate object features and task requirements, has been claimed as first-order motor planning. For example, the hand is opened wider when reaching for larger objects than when reaching for smaller objects. For motor planning in different types of manual action not only object features and immediate task constraint are relevant but also anticipated future states (action goals) are taken into account. This has been called second-order planning. Second-order motor planning is evident, for example, in reaching for an upturned glass to pour something into it and drink from it. The glass is intuitively grasped with an initially uncomfortable hand posture in which the thumb points downward. As a result, after turning the glass, the hand ends up in a final comfortable posture so that there is optimal control over the glass to drink from it comfortably. This toleration of the initial uncomfortable hand posture to end the movement in a comfortable hand posture is known as the *end-state comfort effect* (Rosenbaum et al., 1990). Rosenbaum et al. (1990) investigated the ESC in the laboratory for the first time in their now seminal bar-transport experiment. Participants were asked to grasp a horizontally arranged two-colored wooden bar with their right hand and place it in a vertical position in either a left or right target. One side of the bar was colored black, and the other side of the bar was colored white. If the left side of the bar was to be placed in either the left or right target, largely all participants grasped the bar with an uncomfortable underhand grip. However, when the right side of the bar was to be placed in either target, participants always grasped the bar with a comfortable overhand grip. Regardless of the location of the target, the participants always grasped the bar in such a way that they would end the movement with a comfortable hand posture. The ESC is taken as evidence that the representation of the action goal is anticipated prior to movement initiation, and that this representation has a significant influence on motor planning and the selection of the initial grasp posture (Rosenbaum et al., 2007, 2012). This conclusion is also supported by theories of cognitive psychology which postulate that actions are controlled by the internal representation of action goals and their anticipated features (Hommel et al., 2001; Hoffmann, 2003; Schack, 2004; Schütz-Bosbach and Prinz, 2007). These theories particularly emphasize the close connection between cognition and action. The association between action and its anticipated sensory effects is assumed to be bidirectional.

Numerous studies have previously addressed anticipatory action planning of grasping movements and the ESC in different contexts, such as bimanual actions (Weigelt et al., 2006; Hughes and Franz, 2008), the development of anticipatory action planning (Weigelt and Schack, 2010; Stöckel et al., 2012), the variation of action demands (Hughes et al., 2012; Modersitzki and Studenka, 2020) or in a social context (Herbort et al., 2012). In contrast, there are just a few studies on cognitive aspects such as the representation of grasping actions. Nevertheless, existing studies suggest a manifested representation of grasping actions in memory that favors the comfortable end posture of the hand (Zimmermann et al., 2012; Seegelke and Hughes, 2015).

For example, Zimmermann et al. (2012) asked their participants to decide whether they would grasp a bar with an overhand or underhand grip to reach a specific target position of the bar by pressing a button. The results showed that the indication of how they would grasp the bar was similar to the grip choice during the actual execution of grasping movements from previous studies (Rosenbaum et al., 1990; Rosenbaum and Jorgensen, 1992). This shows that even before the actual movement, it is possible to anticipate how the movement will end with the particular initial grasp posture. It can be concluded that mental representations of future grasp postures can be retrieved quickly and even when the actual movement has not yet been initiated (Rosenbaum et al., 2012).

Another study by Seegelke and Hughes (2015) investigated the influence of action goal and the possibility/impossibility to perform an action sequence on motor imagery of a grasping action. They presented their participants two pictures at the same time with the left image depicting the start position of a right hand holding a bar and with the right image showing the right hand holding the bar at the end of the action sequence. The participants had to do a mental rotation and had to judge as quickly and accurately as possible whether the shown action sequence is physically possible or physically impossible to perform. Results revealed significantly longer reaction times for action sequences depicting a physically impossible to perform action compared to action sequences showing a physically possible to perform action. Additionally, when a 90° and a 135° mental rotation was required, reaction times to final comfortable grasp postures were shorter compared to final uncomfortable grasp postures. This effect only occurred for physically possible to perform action sequences. The authors interpret these results with the fact that a motor representation of grasping actions is manifested in memory in which end-state comfort is prioritized. They suggest that motor representations contain information about the spatio-temporal movement organization as well as the possibility to physically perform an action.

The assumption that motor representations contain information about both the spatio-temporal organization of movement and the possibility of physically performing an action is also supported by Güldenpenning et al. (2012). They examined the cognitive representation of a complex movement using a priming paradigm. The participants were presented with prime images of different states during the approach or flight-phase of a high jump. The subsequent target images also showed different states of the approach or flight-phase of a high jump. Prime-target pairs could therefore represent either the same or different movement phases. In addition, the prime-target pairs could represent the movement in its chronological order (the prime represents an earlier state of the movement than the target) or in its reversed order (the prime represents a later state of the movement than the target). The participants were then asked to decide as quickly and accurately as possible whether the body posture shown in the target image depicted a posture from the approach phase or the flight phase. The results revealed that reaction times were shorter when prime-target pairs reflected the chronological order of the actual high jump (i.e., prime from approach phase, target from flight phase) compared to prime-target pairs that reflected the reversed order (i.e., prime from flight phase, target from approach phase). This means that subjects reacted faster when the prime image depicted an earlier state of the movement than the state in the target. Güldenpenning et al. (2012) conclude that the perception of a static image of an action activates representations of future states of this movement. Accordingly, the prime images led to an anticipation of future movement states and thus facilitated the processing of the target images if these represented a future state of movement (and thus represented a physically possible to perform movement) or to inference the processing of the target images if these did not represent a future state of movement (and thus did represent a physically impossible to perform movement).

In contrast, a recent study by Herbort et al. (2019) suggests that anticipated body movements do not play a role in the planning and selection of grasp postures. In several experiments participants had to grasp and turn a circular knob to rotate a pointer to different targets. While in a pretest and posttest the movement of the pointer corresponded to the movement of the hand and knob, this mapping was modulated in an adaptation phase that included virtual rotations. In this adaptation phase, the mapping between the hand and pointer movements was adjusted in such a way that the same pointer rotation required a larger or smaller hand rotation. Normally, in such a grasping task, it can be observed that, depending on the intended pointer rotation, participants grasp the knob in such a way that they reach end-state comfort. Thus, when final grasp postures are anticipated, manipulating the mapping between the object movement and body movement should affect grasp posture selection. Firstly, as expected, they found that participants adjusted their grasps so that they showed the ESC depending on the rotation of the pointer. However, they also found that the manipulated mapping between pointer and hand movement in the adaptation phase had no influence on the choice of grip posture in the posttest. The authors interpret this finding as evidence that grasp posture planning is not based on the anticipation and representation of body movements. Furthermore, they suggest as a possible alternative explanation that grasps can be adapted to different object manipulations based on the direct cognitive link between the object manipulation task and the associated grasps.

Although numerous studies suggest a link between action (initial grasp posture) and action effect (final grasp posture) in grasping movements (Rosenbaum et al., 1990; Zimmermann et al., 2012), it is still unclear whether the association between an initial grasp posture and its action effect, in form of a final action state (second-order motor planning), is represented in memory. Such an association in memory could be used for anticipatory motor planning and to plan for end-state comfort. While Seegelke and Hughes (2015) provide initial insights into how grasping action sequences are represented in memory by using a motor imagery task, they did not investigate the direct association between two action states in memory. Based on this, the derived research question for the present study was whether the perception of a grasp posture holding a bar leads to a goal-directed activation of the action-related representation of a grasping action.

To answer this research question, a reaction time experiment was conducted using a priming paradigm. A preceding practical task was implemented containing the same grasping action that was used in the priming experiment to familiarize the participants with the grasping action. In the present priming experiment, the bar-transport task (Rosenbaum et al., 1990), which has already been used in numerous experiments studying the ESC, served as the represented grasping action. The subjects were presented with prime images showing the initial grasp postures (comfortable overhand grip and uncomfortable underhand grip) when holding the two-colored bar (white end and black end) in the bar-transport task. Target images represented the final states of this movement (comfortable thumb-up and uncomfortable thumb-down grasp posture). Due to the different grasp postures in the prime and target, the prime and target images in combination thus represented different action sequences as well as physically possible and physically impossible actions. The participants were asked to react to the target image by deciding what color the upper part of the bar was. The grasp posture was therefore irrelevant for that decision. The impossible action should help to interpret the potential priming effects with respect to perceptual and cognitive processes. To find out whether perceptual priming effects of the hand in general played a role in the task (see also Bläsing et al., 2014; Güldenpenning et al., 2015), we implemented additional control conditions in which the bar and hand were presented in the final state of the bar-transport task in both the prime and the target. Thus, prime and target could show a thumb-up or thumb-down grasp posture as well as a bar position with the black top color or the white top color. In these conditions, prime-target pairs were manipulated in a way that the bar position and the grasp posture were either the same (and thus perceptually similar) or different between prime and target.

Studies on grasping behavior show the tendency of people to select an initial grasping posture in anticipation of future action states (e.g., to achieve a comfortable final grasp posture), which leads to the assumption that there is a direct connection in memory between different action states. This view on anticipative motor planning is also postulated by the ideo-motor theory, which assumes a bidirectional association between action and action effect in memory (Hommel et al., 2001; Hoffmann, 2003). Such an association in memory might be the basis to plan actions in an anticipatory way and to plan for end-state comfort. Therefore, the association between an initial grasp posture and its specific action effect should be represented in memory. Based on this, we assume that the perception of an initial grasp posture leads to the activation of future grasp posture, which might facilitate the reaction to such postures.

Thus, we hypothesized the following. Reaction times were expected to be shorter for targets showing a comfortable thumb-up grasp posture compared to an uncomfortable thumb-down grasp posture regardless of whether it was preceded by an initial comfortable overhand grip or an initial uncomfortable underhand grip. Since we assume that the effects are caused by cognitive processes such as action-related anticipation and not by the grasp posture in the target itself or the perceptual similarity between prime and target grasp posture (perceptual priming), faster reactions to comfortable final grasp posture were only expected in the condition with the possible actions but not in the condition with the impossible actions. Another reason for expecting the effects only in the possible condition was the assumption that the motor representation of movements is only present for possible actions (Güldenpenning et al., 2012; Seegelke and Hughes, 2015). Based on this we also expected reaction times would be shorter for prime-target pairs reflecting a possible action than prime-target pairs reflecting an impossible action. However, if the perception of an initial grasp posture does not lead to any activation of action-related representations of a grasping action, no influence of the different prime-target pairs on the reaction times was expected.

For the control condition we expected shorter reaction times for prime-target pairs with congruent bar positions, since participants should react to the top color in the target. Thus, a congruent bar position in the prime should prime the reaction, since prime and target require the same response in this case (response-congruency effect; Kunde et al., 2003). As the influence of perceptual processes of the grasp posture was unclear, no hypotheses for the congruency of the grasp between prime and target could be made at this stage. If perceptual processes played a role in the task, then reactions should be shorter to congruent grasp postures than to incongruent grasp postures (perceptual priming). If the perceptual similarity of the grasp between prime and target played no role then the congruency of the grasp should have no influence on the reaction times.

## 2 Method

### 2.1 Participants

To determine the required sample size, a sample-size analysis was carried out in advance using G*Power 3.1 (Faul et al., 2007). Based on a pilot study with *N* = 6, in which an effect size of *ηp^2^* = 0.12 was calculated for the interaction in our 2 × 4 repeated measures design, the results of the sample-size analysis suggest a sample size of at least *N* = 22 participants (given *f* = 0.25, *α* = 0.05, 1-β = 0.95). In total, thirty (*N* = 30) individuals (mean age 25.9 years, *SD* = 3.56, age range 20–34, twenty men, ten women) participated in the study. Using a German version of the modified Edinburgh Handedness Inventory (Oldfield, 1971), all subjects could be classified as right-handed (mean laterality index = 96.67, *SD* = 8.49). All participants reported no visual impairment or corrected visual impairment with glasses or contact lenses, and no motor or cognitive impairments. Participation in the study was voluntary and there was no financial compensation. There was an opportunity for sport students at Bielefeld University to receive course credits for participation. The study was approved in advance by the ethics committee of Bielefeld University. The subjects were also informed about the research procedure before the experiment was conducted and all participants gave their written informed consent following the Declaration of Helsinki.

### 2.2 Apparatus, stimuli, and design

#### 2.2.1 Practical bar-transport task

**Figure 1:**
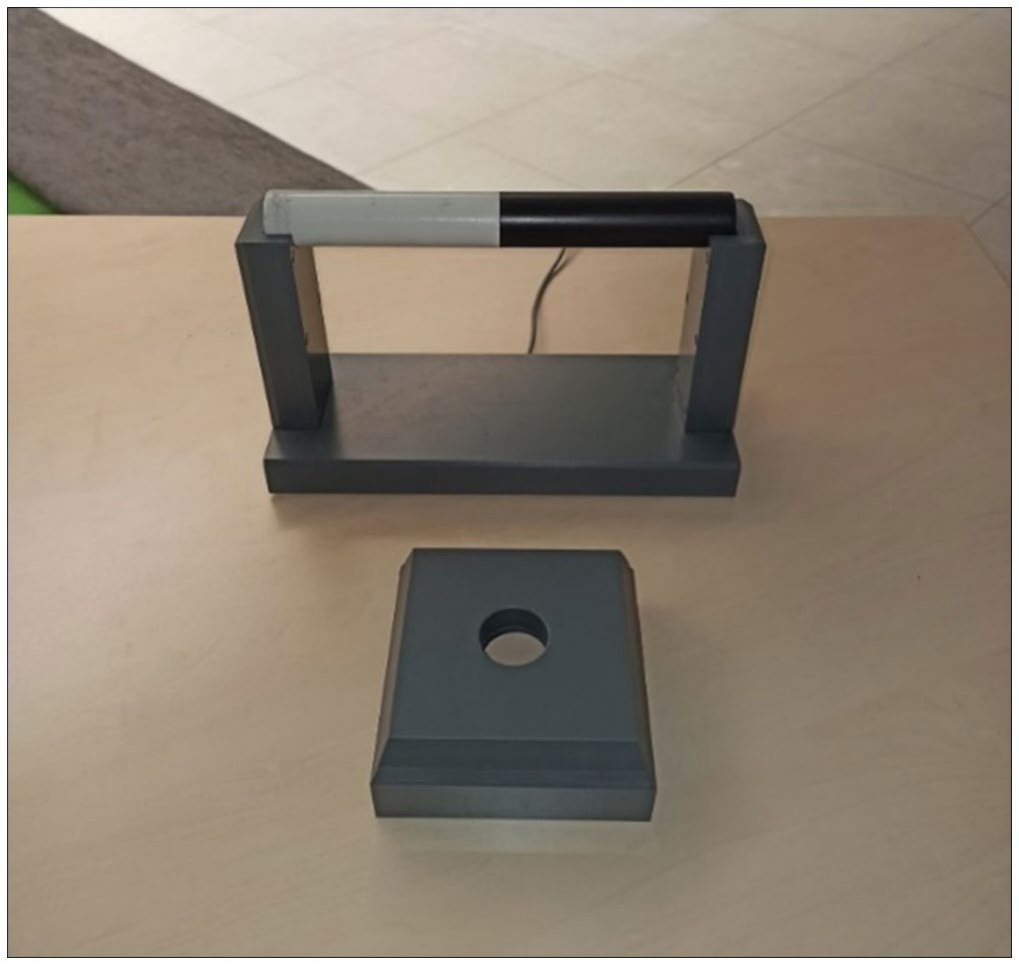
Setup of the bar-transport task. In the start position the two-colored bar rested horizontally in the cradle. The target platform, into which the bar had to be inserted vertically, was placed in front of it.

The setup of the practical bar-transport task was based on the setup of Rosenbaum et al. (1990), except that only one start and one target device was used (see Figure 1).

A two-colored (half white and half black) wooden bar with a length of 25 cm and a diameter of 3 cm was placed horizontally in a cradle. The bar was 16 cm above the table in this starting position. The target in the form of a cube with a length and width of 12.5 cm and a height of 5 cm was placed 10 cm in front of the cradle. The target had a hole in the center with a diameter of 3.5 cm into which the ends of the bar fit.

#### 2.2.2 Reaction time experiment

A computer with a 22-inch screen and the software program PsychoPy (version 3.6.6) were used for the stimulus presentation. The screen had a refresh rate of 60 Hz and a resolution of 1,680 × 1,050 pixels and was placed approximately 60 cm away from the subject. Images were presented and responses and reaction times were recorded using the software (PsychoPy). With the respective index fingers of the left or right hand, the subjects had to enter the responses by pressing the “A” or “L” key on the keyboard. The response assignment of the keys was counterbalanced between subjects. The distance between the two keys was 15.5 cm, measured from the center of the keys.

For the experimental condition, four different images were presented as primes. These images showed a right hand grasping a bar. The bar was lying horizontally in a cradle, similar to the start position in the bar-transport task (Rosenbaum et al., 1990). The bar was colored half black and half white. The prime images depicted either an overhand grip (comfortable) or an underhand grip (uncomfortable). Both grasp postures could be represented with two different start positions of the bar (white left or black left), resulting in four different prime images.

The target images also consisted of four different images in which a right hand holds a bar that is vertically placed in a target, like the end position in the bar-transport task. The images showed either grasping the bar with a thumb up (comfortable) or thumb down (uncomfortable) grasp posture. Both grasp postures (thumb up and thumb down) were possible with either bar position (white on top or black on top).

**Figure 2:**
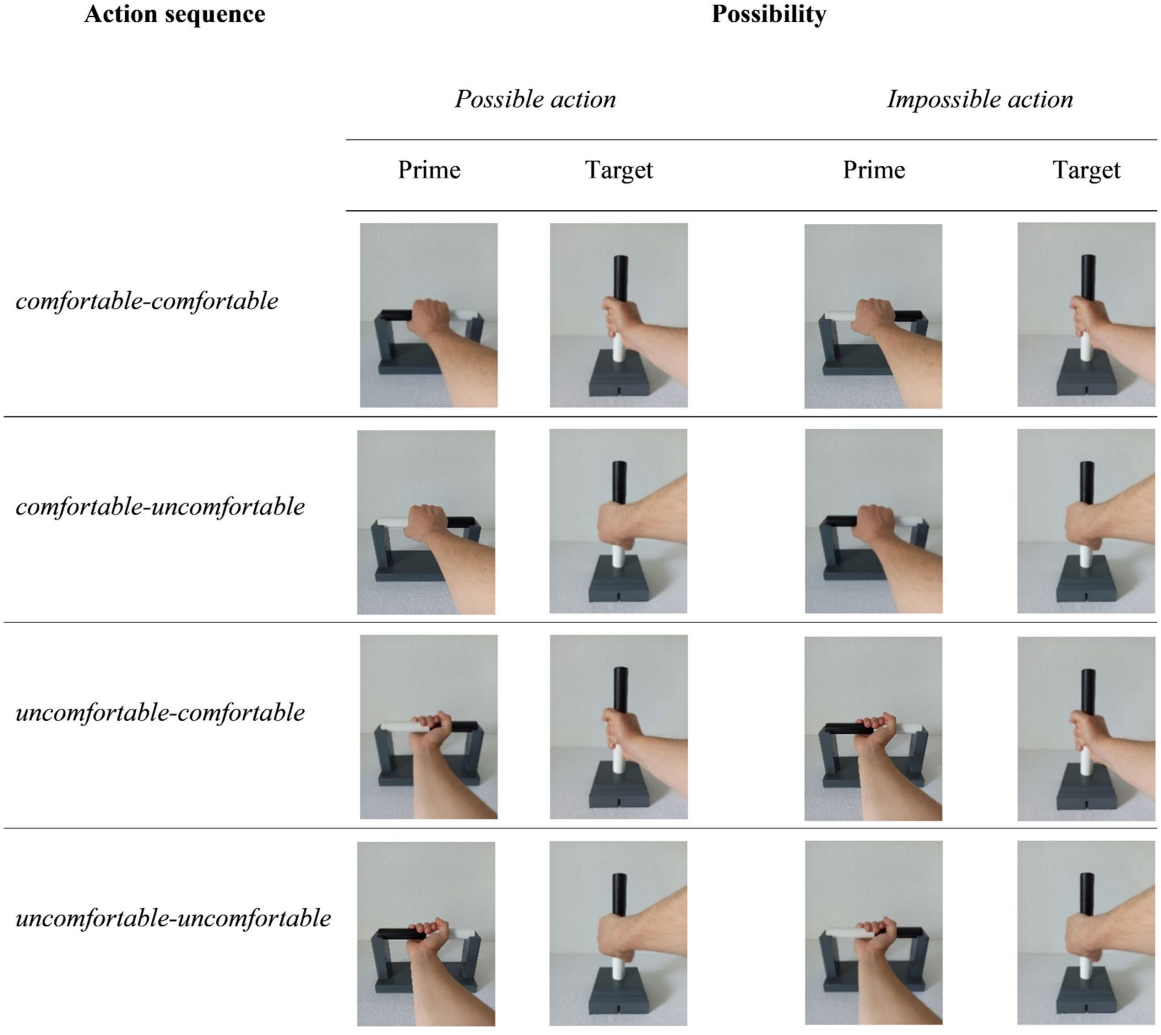
Exemplary prime-target pairs of the experimental conditions. In the prime as well as the target, comfortable or uncomfortable grasp postures could be represented, resulting in four different sequences of actions. The action shown could represent a physically possible to perform as well as impossible to perform action. Due to the different possibilities of the bar position, each condition consisted of two different prime-target pairs. In this figure only the prime-target pairs with the black color on top in the target are presented as an example.

Each prime image was combined with each target image, resulting in a total of 16 different prime-target pairs in the experimental condition. Since both the prime and target images showed comfortable and uncomfortable grasp postures, this resulted in a total of four action sequences (comfortable-comfortable, comfortable-uncomfortable, uncomfortable-comfortable, uncomfortable-uncomfortable). These action sequences represent both possible and impossible actions. For example, an impossible action would be when the prime showed a comfortable overhand grip, and the left side of the bar being black, and the target image showed a comfortable thumb up grasp posture with the white side on top (see Figure 2). Performing this action is not possible without adjusting the grip. The prime-target pairs representing an impossible action were included in the design to be able to draw conclusions about whether the possibility of being able to perform an action had an influence in the processing of the images. Likewise, these were intended to provide information about whether possible priming effects were action-related or solely due to the grip posture in the target. The prime-target pairs of the experimental condition resulted in a 2 × 4 design with the main factors possibility (*possible action, impossible action*) and action sequence (*comfortable-comfortable, comfortable-uncomfortable, uncomfortable-comfortable, uncomfortable-uncomfortable*). Due to the different prime-target combinations, each condition consisted of two different prime-target pairs. Figure 2 shows an example prime-target pair for each experimental condition.

**Figure 3:**
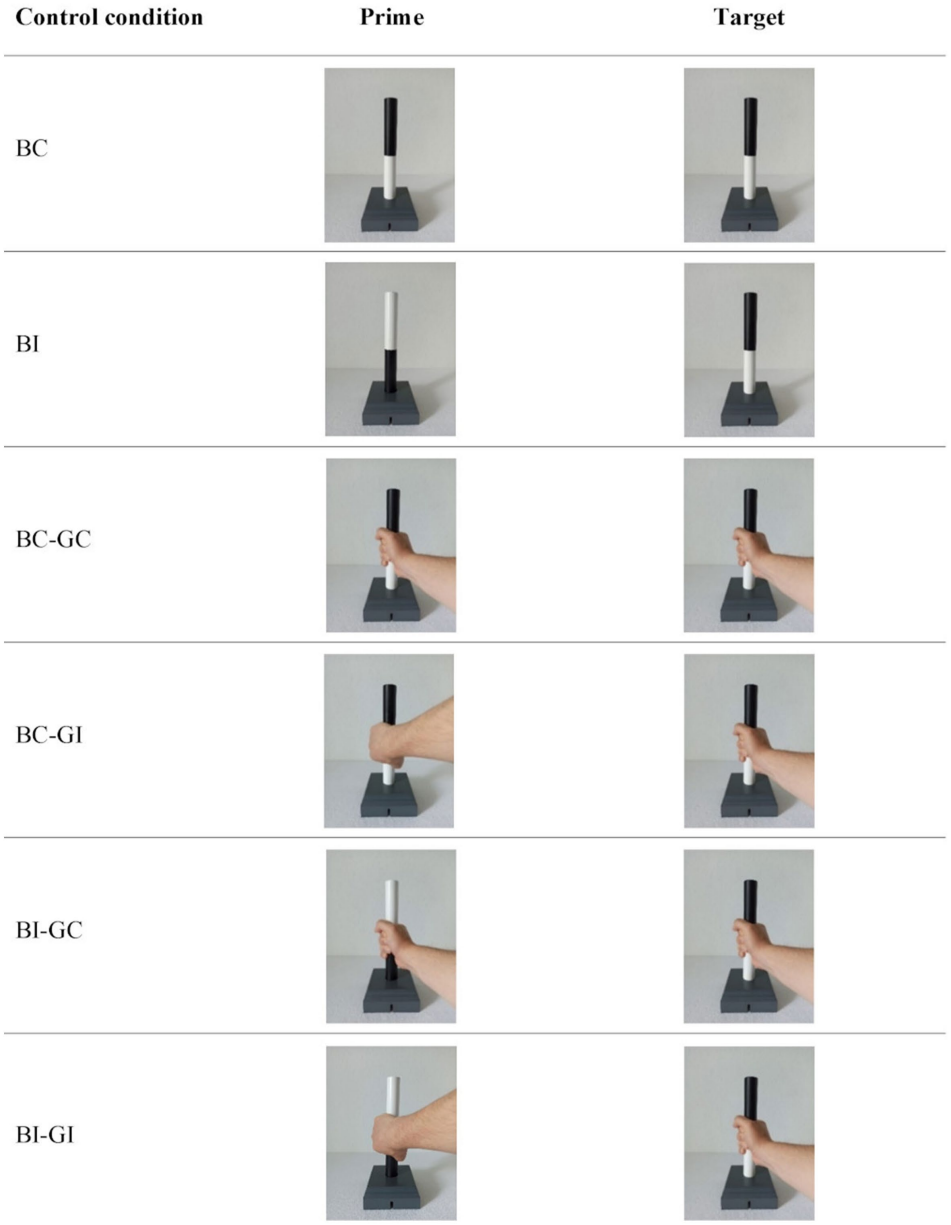
Exemplary representation of a prime-target pair for each of the control condition. Prime and target could have a congruent (BC = bar congruent) or incongruent (BI = bar incongruent) bar position. Similarly, the grip could be either congruent (GC = grip congruent) or incongruent (GI = grip incongruent). Thus, the combination of the prime and target pictures without hand resulted in the conditions BC and BI and the combination of the pictures with hand resulted in the conditions BC-GC, BC-GI, BI-GC and BI-GI. Each condition without hand consisted of two and each condition with hand of four different prime-target combinations. In this figure only the prime-target pairs with the black color on top and a thumb-up grasp posture in the target are presented as an example.

In addition, there were control conditions that aimed to draw conclusions about priming effects due to perceptual features of the grasp postures (see also Bläsing et al., 2014; Güldenpenning et al., 2015). There were control conditions without and with hand shown (see Figure 3). The control conditions with hand were included to test whether the perceptual features (i.e., the perceptual similarity) of different grip types had an influence on the response to the target image. These results should provide information on whether possible priming effects in the experimental conditions were due to perceptual effects (e.g., grasp posture in the prime and target, and thus the perceptual properties, are more similar, which is why responses to the target were faster or slower). In addition, there were control conditions without hand, which were compared with the control conditions with hand in order to find out whether the perception of the hand *per se* had an influence on responses (regardless of whether prime and target had the same perceptual features with regard to the grasp posture). In the control conditions without hand, the prime and target images showed the bar vertically in the target position. There were two different images for both the prime and the target, resulting from the two possible positions of the bar in the target device (white on top and black on top). The combination of the two prime and two target images resulted in four different prime-target pairs. Therefore, the bar position between prime and target could be either congruent (BC) or incongruent (BI; *cf.* Figure 3). Due to the different prime-target combinations, each condition consisted of two different prime-target pairs. The control conditions with hand, in both the prime and target images, depicted the bar vertically placed in the target while a right hand holds the bar. The grasp posture could represent a thumb-up (comfortable) or thumb-down (uncomfortable) posture. Thus, four different prime images and four different target images were obtained. For the control conditions with hand the four prime and four target images resulted in 16 prime-target pairs. Prime and target could be either congruent (BC) or incongruent (BI) with respect to the bar position and congruent (GC) or incongruent (GI) with respect to the grasp posture. This resulted in four different conditions (BC-GC, BC-GI, BI-GC, BI-GI). Due to the different prime-target combinations, each condition consisted of four different prime-target pairs. As an example, one prime-target pair for each condition is shown in Figure 3.

The presented images had a size of 24.3 × 32.3 cm (788 × 1,050 pixels). The images were taken on a white table in front of a white wall to avoid irritating background colors. The action depicted is from a first-person perspective. All images were presented centrally on the screen with a black background.

### 2.3 Procedure

Before the actual reaction time experiment was conducted, subjects completed the bar-transport task to get familiar with the apparatus and movement as well as to have performed the movements once in a practical manner. The cradle of the bar-transport task setup was placed within arm’s reach in front of the subject on the experimental table. The black and white colored wooden bar was placed in the cradle (*cf.* Figure 1). The subject’s task was to grasp the bar with the right hand, lift it out of the cradle and insert it vertically into the target device. Subsequently, the hand was to be placed on the table and the experimenter placed the bar back into the cradle. Before each trial, subjects were instructed how to grasp the bar and which color of the bar to point up at the end. The bar could be grasped with either an overhand or underhand grip and the top color of the bar could be either white or black at the end of the movement. Due to the different possible starting positions of the bar (white left or black left), each action sequence (combination of initial grip and final bar position) was performed twice. Thus, the practical execution of the bar-transport task consisted of eight trials. For each subject, the order of the movements to be performed was randomized.

For the reaction time experiment, subjects sat 60 cm in front of the screen. They were instructed in advance in written form that two pictures would be shown on the screen one after the other at a short time interval. They were asked to respond to the second image as quickly and as accurately as possible to the question „Ist die obere Farbe des Stabs schwarz? “(German for “Is the top color of the bar black?”). One of the two keys on the keyboard (A and L) represented the answer “yes” and the other “no.” The key assignment was counterbalanced between subjects.

**Figure 4:**
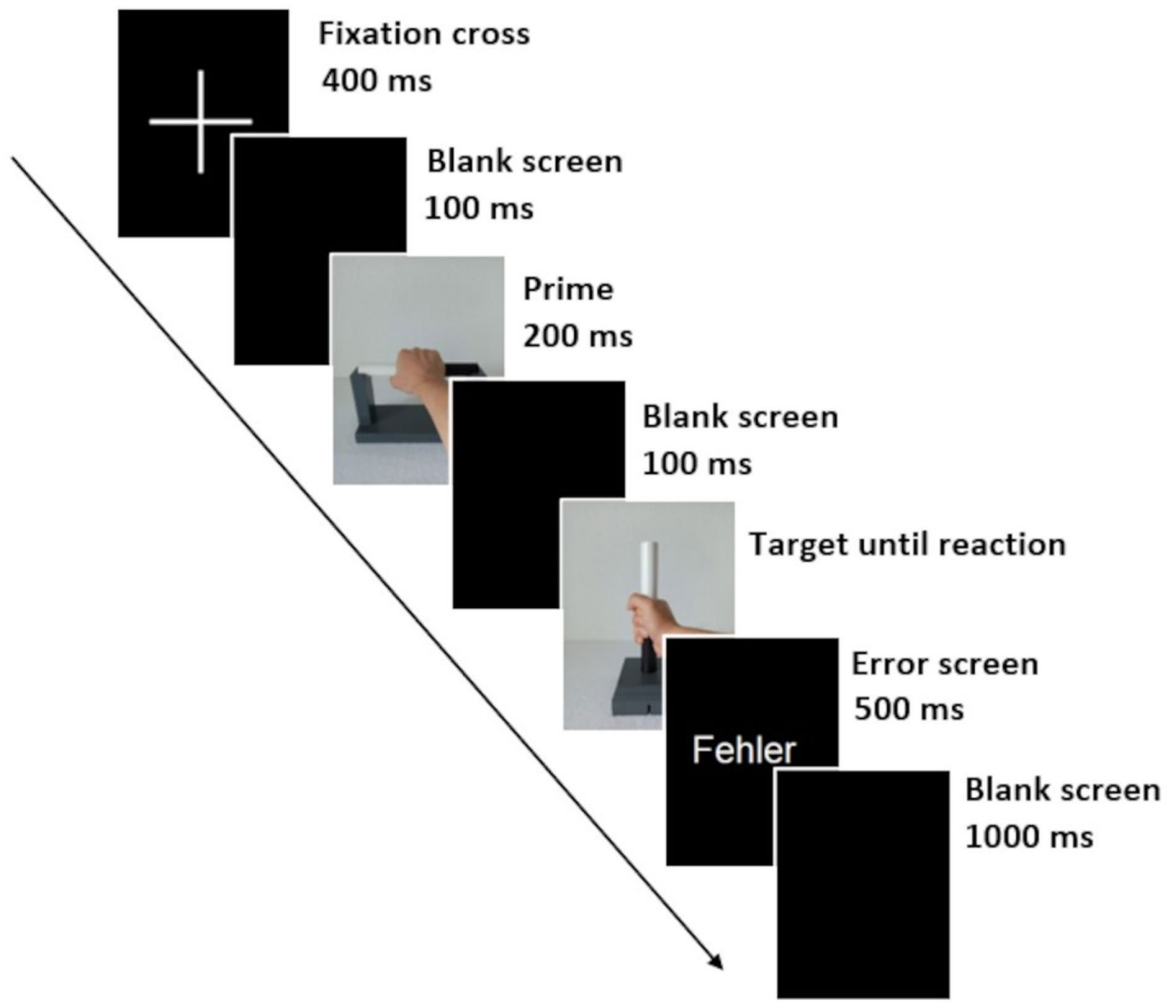
The procedure of a trial with a wrong answer. A trial began with the presentation of a fixation cross for 400 ms. A black screen (blank screen) shown for 100 ms was followed by the presentation of the prime picture for 200 ms. The presentation of another blank screen for 100 ms was followed by the target picture until the response. If the response was incorrect, an error screen with the message “Fehler” (German for “error”) first appeared for 500 ms, followed by a blank screen for 1,000 ms (if the response was correct, only a blank screen appeared for 1,500 ms). This was followed by the fixation cross of the next trial. The exemplary prime-target pair represents the action sequence comfortable-comfortable within the possible action.

Each trial started with the presentation of a fixation cross centered on the screen for 400 ms. This was followed by a black screen (blank screen) for 100 ms, a prime image shown for 200 ms, a second blank screen for 100 ms, and the target image, which was shown until the response key was pressed. If the answer was correct, a blank screen appeared for 1,500 ms before the next trial began. If the answer was incorrect, the word “Fehler” (German for “error”) appeared in the center of the screen for 500 ms, followed by a blank screen for 1,000 ms. The procedure within a trial and the times of stimulus presentation were designed based on priming studies from the literature (Güldenpenning et al., 2012; Bläsing et al., 2014; Decroix and Kalénine, 2018) and results of a preceding pilot study with six subjects. The procedure within a trial with a wrong answer is shown in Figure 4.

When starting with the experimental blocks, a practice block with the images of the experimental condition took place in advance. Each prime-target pair of these conditions was shown once, resulting in 16 practice trials. Data from these trials were not analyzed. This practice block was followed by the experimental blocks consisting of the experimental conditions. Throughout the experiment, each prime-target pair of the experimental condition was shown 20 times, resulting in a total of 40 trials per experimental condition (because each experimental condition consisted of two prime-target pairs). The blocks were divided in such a way that each prime-target pair was presented five times per block. This resulted in four experimental blocks of 80 trials each. The four experimental blocks were followed by a practice block using the images from the control conditions. Each prime-target pair of the control conditions (with hand and without hand) was shown once, resulting in 20 practice trials. In the subsequent control blocks, each prime-target pair was presented ten times. Each control condition without hand was thus shown 20 times and each control condition with hand 40 times. This was divided into two control blocks in which each prime-target pair was shown five times, resulting in 100 trials per control block. After each block, the subjects were given the opportunity to take an individually long break and could independently start the next block by pressing the space bar. Half of the participants started the experiment with the experimental blocks, the other half started with the control blocks.

The presentation of prime-target pairs was randomized within blocks. In total, the experiment consisted of 36 practice trials and 520 experimental and control trials. The duration of the reaction time experiment averaged approximately 30 min.

### 2.4 Data analyses

For the analysis of the data, the reaction time (in ms) was used, which defines the time between the appearance of the target stimulus and pressing the response button. For a holistic analysis of the data, the average error rates (in %) of the conditions were also examined. All responses that were faster than 100 ms and slower than 1,000 ms (0.96% of the data) were considered anticipations and outliers, respectively, and were therefore not included in the analysis (see also Alhaj Ahmad Alaboud et al., 2016; Güldenpenning et al., 2019; Ni et al., 2019). Trials with incorrect responses (2.88% of the data) were excluded from the analysis of reaction times. The mean values of reaction times (in ms) and error rates (in %) for each subject were then calculated for each condition. Statistical analysis was performed using IBM SPSS Statistics (version 28.0.1.0).

To measure an influence of the within-subject factors *possibility* (possible action, impossible action) and *action sequence* (comfortable-comfortable, comfortable-uncomfortable, uncomfortable-comfortable, uncomfortable-uncomfortable) on the dependent variable reaction time and error rates, repeated measures ANOVA were performed. Subsequently, to check whether the factor *action sequence* within the conditions possible action and impossible action had an influence on the dependent variable, two separate repeated measures ANOVA were performed.

To measure the influence of the factor *control condition* (BC, BI, BC-GC, BC-GI, BI-GC, BI-GI), repeated measures ANOVA were performed for the dependent variable reaction time and error rates.

If the sphericity of the data was not given, the Greenhouse–Geisser correction was used. In this case, the corrected significance level and the corrected degrees of freedom were reported. For multiple comparison, Bonferroni-corrected paired t-tests were performed, and the reported significance values (*p*-value) correspond to the corrected value by this correction. A significance level of *α* = 0.05 was used for all statistical tests and all statistical values, except for significance values, were rounded to two decimal places.

## 3 Results

### 3.1 Reaction times of the experimental conditions

The repeated measures ANOVA with the within-subjects factors *possibility* and *action sequence* revealed no significant effect on reaction time for the main factor *possibility*, *F*(1, 29) = 0.03; *p* = 0.869; *ηp^2^* = 0.00. Additionally, the main factor *action sequence* showed no significant effect, *F*(3, 87) = 0.84; *p* = 0.476; *ηp^2^* = 0.03. In contrast, a significant interaction effect was found for the main factors *possibility* and *action sequence*, *F*(1.86, 53.97) = 4.00; *p* = 0.027; *ηp^2^* = 0.12.

**Figure 5:**
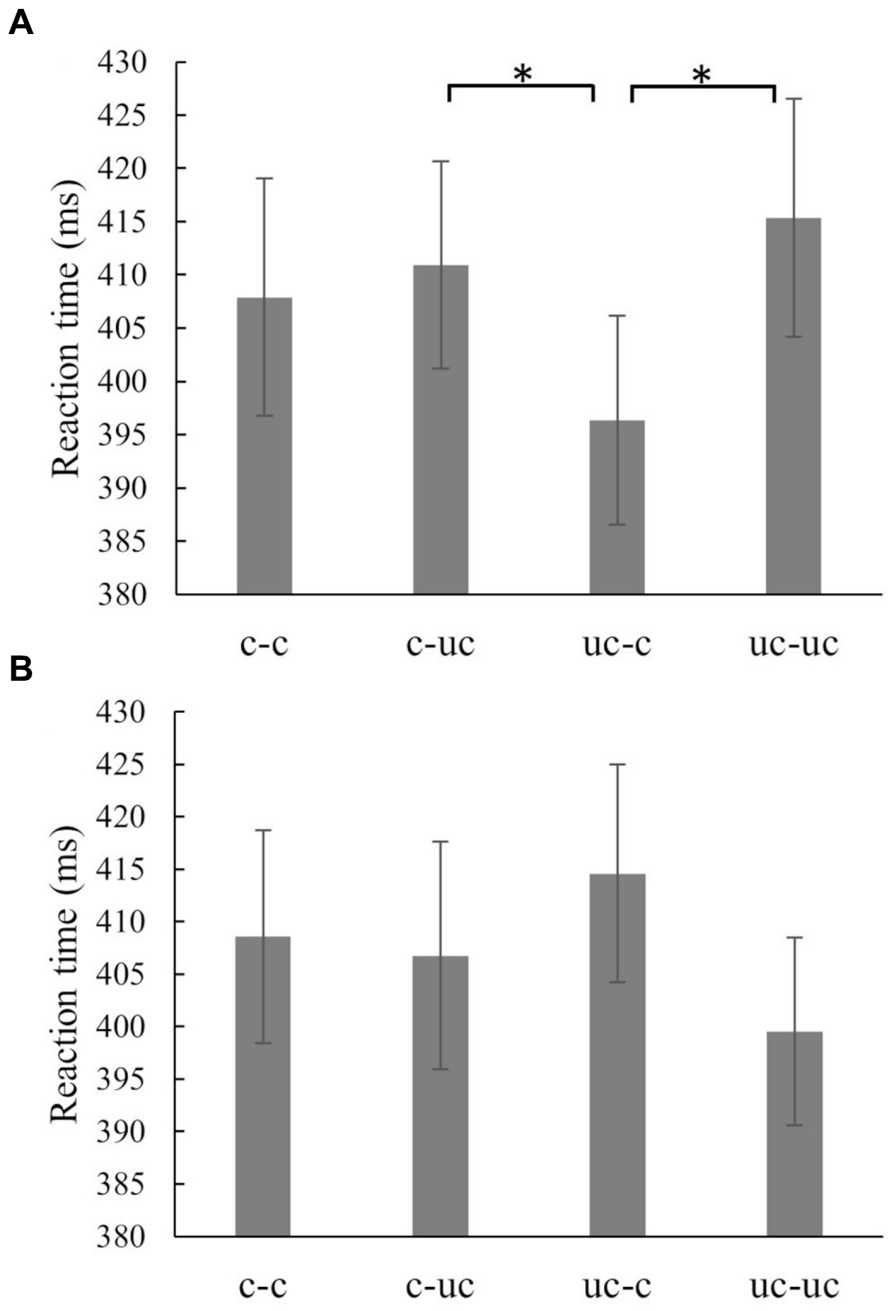
Reaction times of the experimental conditions. Reaction times of the action sequences c-c (comfortable-comfortable), c-uc (comfortable-uncomfortable), uc-c (uncomfortable-comfortable) and uc-uc (uncomfortable-uncomfortable) within the possible action condition **(A)** and impossible action condition **(B)**. Reaction times (in ms) are shown, with the error bar indicated as ± standard error of the mean (in ms). The “*” stands for *p* = < 0.05.

The subsequent repeated measures ANOVA within the possible action condition was able to measure a significant main effect of the *action sequence*, *F*(1.92, 55.79) = 4.19; *p* = 0.021; *ηp^2^* = 0.13. Paired *t*-tests with Bonferroni correction between all four action sequence conditions showed significantly shorter reaction times of the uncomfortable-comfortable condition (*M* = 396.32 ms, *SD* = 53.71 ms) compared to the uncomfortable-uncomfortable condition (*M* = 415.34 ms, *SD* = 61.33 ms), *t*(29) = −3.03; *p* = 0.031; *d* = 0.55. Accordingly, after a prime with an uncomfortable underhand grip, reactions to targets with a comfortable thumb-up grasp posture were 19.02 milliseconds faster compared to an uncomfortable thumb-down grasp posture. Additionally, the reaction times of the uncomfortable-comfortable condition (*M* = 396.32 ms, *SD* = 53.71 ms) turned out to be significantly shorter than those of the comfortable-uncomfortable condition (*M* = 410.91 ms, *SD* = 53.33 ms), *t*(29) = 3.18; *p* = 0.021; *d* = 0.58. When a prime with an uncomfortable underhand grip was followed by a comfortable thumbs-up grasp posture presented in the target, responses were 14.59 milliseconds faster compared to a prime with an overhand grip followed by an uncomfortable thumbs-down grasp posture presented in the target. Figure 5A illustrates the reaction times for the different action sequences within the possible action condition.

The repeated measures ANOVA within the impossible action condition failed to measure a significant main effect of the *action sequence*, *F*(2.16, 62.53) = 2.72; *p* = 0.070; *ηp^2^* = 0.09. Interestingly, the descriptive results indicate that responses following an underhand grip show a reversed pattern compared to the possible condition. In the impossible condition, faster mean responses were measured for the uncomfortable-uncomfortable (*M* = 399.53 ms, *SD* = 49.03 ms) condition compared to the uncomfortable-comfortable (*M* = 414.75 ms, *SD* = 56.84 ms) condition. The reaction times for each action sequence condition within the impossible action condition are shown in Figure 5B.

### 3.2 Error rates of the experimental conditions

The repeated measures ANOVA with the within-subjects factors *possibility* and *action sequence* revealed no significant effect on error rates for the main factor *possibility*, *F*(1, 29) = 1.15; *p* = 0.293; *ηp^2^* = 0.04. The main factor *action sequence* also had no significant effect on error rates, *F*(3, 87) = 1.37; *p* = 0.258; *ηp^2^* = 0.05. In contrast, a significant interaction effect was found for the factors *possibility* and *action sequence*, *F*(3, 87) = 2.95; *p* = 0.037; *ηp^2^* = 0.09.

Additionally, repeated measures ANOVA with the factor *action sequence* within the conditions possible action and impossible action were conducted. The repeated measures ANOVA with the factor action sequence within the possible action failed to measure an effect on the error rate, *F*(3, 87) = 1.08; *p* = 0.363; *ηp^2^* = 0.04. Within the possible action, the different action sequences therefore did not differ statistically in terms of the error rates. In contrast, the repeated measures ANOVA with the factor action sequence within the impossible action was able to determine a significant influence on the error rate, *F*(2.40, 69.62) = 3.73; *p* = 0.022; *ηp^2^* = 0.11. Subsequent Bonferroni-corrected *t*-tests, however, were unable to measure any significance in the pairwise tests (all *p’s* > 0.05). The lowest significance level was reached with *p* = 0.068 between the uncomfortable-comfortable (3.92%) and uncomfortable-uncomfortable (1.75%) conditions. The subjects made errors less frequently in the uncomfortable-uncomfortable condition, even though the pairwise comparison was not significant. The different action sequences within the impossible action therefore did not differ statistically in terms of the error rates. The error rates of all action sequences within the possible as well as impossible condition are shown in Supplementary Table S1.

### 3.3 Reaction times of the control conditions

**Figure 6:**
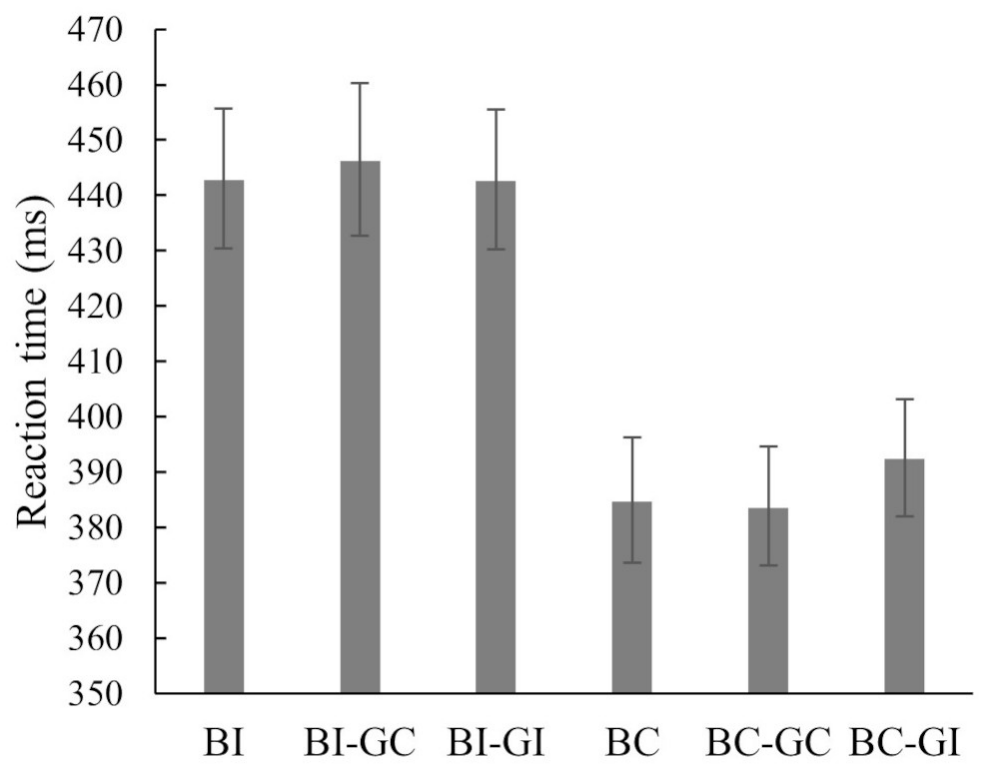
Reaction times of the control conditions. Shown are the reaction times (in ms) with the error bar indicated as ± standard error. Each of the condition depicting a congruent bar position (BC, BC-GC, BC-GI) reached significant shorter reaction times (*p* = < 0.001) than each of the condition depicting an incongruent bar position (BI, BI-GC, BI-GI) no matter if the grasp posture between prime and target was congruent or not.

The repeated-measures ANOVA for the within-subjects factor *control condition* (BC, BI, BI-GC, BI-GI, BC-GC, BC-GI) showed a significant effect on reaction time, *F*(3.46, 100.26) = 62.06; *p* = < 0.001; *ηp^2^* = 0.68. The reaction times of each of the conditions BC (*M* = 384.89 ms, *SD* = 61.80 ms), BC-GC (*M* = 383.85 ms, *SD* = 58.84 ms), and BC-GI (*M* = 392.57 ms, *SD* = 57.94 ms) were significantly shorter (all *p’s* < 0.001) than the reaction times of conditions BI (*M* = 443.02 ms, *SD* = 69.21 ms), BI-GI (*M* = 442.85 ms, *SD* = 68.87 ms), and BI-GC (*M* = 446.48 ms, *SD* = 75.46 ms; *cf.* Figure 6), respectively. All significant pairwise comparisons between the control conditions and the statistical values are shown in Table 1. Overall, the results for all control conditions show faster responses to prime-target pairs where the bar position is congruent, regardless of whether the grasp posture between prime and target is congruent or incongruent or whether a grasp is shown or not.

**Table 1:** Overview of the comparison of significant different control conditions.

		BC	BC-GI	BC-GC
	Reaction time (ms)	384,89	392,57	383,58
BI	443,02	*t*(29) = 10.55 *p* = < 0.001 *d* = 1.93	*t*(29) = 8.28 *p* = < 0.001 *d* = 1.51	*t*(29) = 9.74 *p* = < 0.001 *d* = 1.78
BI-GI	442,52	*t*(29) = 10.72 *p* = < 0.001 *d* = 1. 96	*t*(29) = 8.56 *p* = < 0.001 *d* = 1.56	*t*(29) = 10.48 *p* = < 0.001 *d* = 1.91
BI-GC	446,48	*t*(29) = 9.54 *p* = < 0.001 *d* = 1.74	*t*(29) = 7.26 *p* = < 0.001 *d* = 1.33	*t*(29) = 10.47 *p* = < 0.001 *d* = 1.91

### 3.4 Error rates of the control conditions

The repeated measures ANOVA for the factor *control condition* was able to determine a significant influence on the error rates, *F*(3.01, 87.39) = 3.02; *p* = 0.034; *ηp^2^* = 0.09. However, the post-hoc tests subsequently performed with Bonferroni-correction showed no significant differences between the individual control conditions. Only the comparison between conditions BC and BI approached the significance level of 0.05 with *p* = 0.069, whereby the error rate in the BC condition (1.17%) was lower than in the BI condition (4.17%). The control conditions therefore did not differ statistically in the error rates. The error rates of all control conditions are shown in Supplementary Table S2.

## 4 Discussion

The present study used a priming paradigm to investigate whether the visual perception of a grasp posture holding a bar leads to the goal-directed activation of action-related representations of grasping actions. For that purpose, prime images were shown representing an initial grasp posture holding a bar whereas target pictures were shown representing the final grasp posture of this grasping action. The participants were asked to react to the top color of the bar shown in the target image, whereby the grasp posture was irrelevant for this decision. Within the possible action, it was shown that after the presentation of an uncomfortable underhand grip, responses to comfortable thumb-up grasp postures were faster than to uncomfortable thumb-down grasp postures. However, the presentation of a comfortable overhand grip had no effect on the responses to subsequent comfortable thumb-up and uncomfortable thumb-down grasp postures in the target. No significant effect of the factor action sequence could be determined for the impossible condition. In addition, the results showed, contrary to expectation, that responses to prime-target pairs representing impossible and impossible actions were equally fast.

The analysis of the prime-target pairs within the possible action revealed a significant effect of the action sequence on the reaction times. The reaction times after the presentation of an underhand grip were shorter for target pictures with a thumb-up gasping posture compared to target pictures with a thumb-down gasping posture. In contrast, there was no effect for the action sequence on error rates. The interpretation and attempt to explain the general deviation of the error rates from the reaction times in this study will be made at a later stage. For this reason, the following comparison with existing studies and the interpretation of the results is only carried out based on response times. Compared to the underhand grip, the presentation of an overhand grip did not lead to different reaction times between target images depicting a thumb-up and a thumb-down grasp posture. The error rates also showed no differences between responses to target images with a thumb-up and thumb-down grasp posture. The results of the present study partially match the results of Seegelke and Hughes (2015), who showed a faster motor imagery of grasping actions with a comfortable thumb-up end posture of the hand compared to an uncomfortable thumb-down end posture. However, they did not differentiate between different initial grasp postures, which means that a comparison with this study is only possible to a limited extent here. In addition, the subjects in this study had to mentally rotate the movement and make a decision based on this, focusing on the grasping action. In the present study, the movement was irrelevant for the decision-making task and no mental rotation was necessary for the reaction to the target picture. The partially deviating results of the present study from the results of Seegelke and Hughes (2015) could therefore be due to different paradigms used (priming paradigm and mental rotation task) and a different analysis (separation according to initial grasp postures and no separation according to initial grasp postures). Likewise, the results only partially match studies using a bar-transport paradigm in which the actual grasping behavior was examined (Rosenbaum et al., 1990; Hughes et al., 2012) or in which participants were asked to indicate how they would grasp the bar without performing this movement (Zimmermann et al., 2012). In these studies, an initial underhand grip or an initial overhand grip was selected in order to end the movement with a comfortable end posture of the hand. However, it should be emphasized, that the priming effect of an underhand grip fits well with studies on ESC using the bar-transport paradigm (Rosenbaum et al., 1990; Hughes et al., 2012), which showed that an uncomfortable initial grasp posture (underhand grip) was used to end the movement in a comfortable final (thumb-up) grasp posture.

Based on the results, we assume that the perception of an underhand grip in the prime led to the activation of an action-related representation of future states of a grasping action (Schütz-Bosbach and Prinz, 2007; Urgesi et al., 2010; Güldenpenning et al., 2012). A perceived underhand grasp thus led to the activation of the representation of a grasping action with a comfortable end posture of the hand (ESC). In other words, it can be assumed that the participants anticipated future action goals after perceiving the underhand grip, which led to a facilitation of the target images on which the action effect of a comfortable final grasp posture was represented. This action-related priming-effect suggests that an anticipated (cognitive) link between the two action stages is represented in memory. The underhand grip is thus associated with a certain action effect, in this case with an action effect that is consistent with the ESC. This fits with the assumption that motor representations contain information about the spatio-temporal movement organization and that a representation of grasping movements is established across the lifespan that is consistent with the ESC (Seegelke and Hughes, 2015). We assume that the connection between different action states in memory that we found in our study could be relevant for the anticipatory planning of action. This assumption is supported by cognitive psychological theories assuming that actions are represented in memory and controlled based on their intended effects they produce (Hommel et al., 2001; Hoffmann, 2003; Schack, 2004).

In comparison to the underhand grip, it can be assumed that the perception of an overhand grip did not lead to any activation of action-related representations of grasping actions. As the result is unexpected and the results do not allow any further interpretation, an explanation is only possible to a limited extent. One possible reason could be the different rotation options offered by an overhand grip and an underhand grip to solve the present movement task. With an underhand grip, both the final thumb-up grasp posture (90° rotation) and the thumb-down grasp posture (270° rotation) can only be achieved by pronating the wrist. With an overhand grip, on the other hand, a supination (90° rotation) is required to achieve the final thumb-up grasp posture and a pronation (90° rotation) is required to achieve the final thumb-down grip position. In addition, the overhand grip represents a standard grip choice of the habitual system, while the underhand grip is selected by the goal-directed system in order to achieve a specific action goal, such as a comfortable end posture (Stöckel et al., 2012). The planning and execution of goal-directed actions (goal-directed system) appear to be more difficult, involve increased cognitive effort and differ at the neuronal level from grasping actions of the habitual system (Westerholz et al., 2014; Zhang et al., 2020). It is possible that the perception of an underhand grip led to increased cognitive activation compared to the overhand grip. This could be the reason why we found a priming effect for the underhand grip but no priming effect for the overhand grip. As our results do not provide enough information to explain the different results of the overhand grip and the underhand grip, further research is needed to investigate the possible reasons.

Importantly, no significant effect of action sequence on reaction time could be measured in the impossible action condition. Although a significant main effect of action sequence on error rates was measured, the post-hoc comparison did not survive the Bonferroni correction. The results of the impossible condition suggest that an underhand grip was not automatically followed by shorter reaction times to target pictures that depicted an action goal with a comfortable thumb-up grasp posture, regardless of the physical possibility to perform an action. The different results of the possible action condition and impossible action condition are supported by studies that show that the awareness of whether an action is physically possible to perform is taken into account in the processing of action sequences (Güldenpenning et al., 2012; Seegelke and Hughes, 2015). For example, participants in Seegelke and Hughes (2015) showed a faster motor imagery for action sequences with a comfortable final grasp posture when the action shown was possible to perform, but no differences in responses to comfortable and uncomfortable final grasp postures when the action shown was impossible to perform.

Interestingly, the descriptive results for the reaction times and error rates of the impossible condition indicate that the reactions after an underhand grip show a reversed pattern compared to the possible condition. Participants responded faster and more accurate to uncomfortable than to comfortable final grasp postures after a shown underhand grip when the shown action sequence depicted an impossible action. A possible post-hoc explanation could be that subjects used the perceived grasp posture and bar orientation in the prime to anticipate the final bar orientation. The anticipated bar orientation represents the final bar orientation that would have been reached if the end-state comfort effect would have been reached. In the possible condition, for an underhand grip in the prime, this is the bar orientation that is reached with a comfortable thumb-up grip, i.e., a 90° counterclockwise rotation of the bar. In the impossible condition, for an underhand grip in the prime, a 90° counterclockwise rotation of the bar is reached with an uncomfortable thumb-down grasp posture in the target. Thus, in both cases, when an underhand grip is perceived, the right color of the bar is expected to be the top color in the target, as this represents the final orientation of the bar with a movement that satisfies the end-state comfort. This alternative explanation of the results would lead to the conclusion that participants anticipated final bar orientations rather than final grasp postures. Thus, an initial grasp posture and object orientation is associated with an object movement. This fits with a recent study by Herbort et al. (2019) which suggests that the anticipation of hand movements does not play a role in the planning of grasping actions. However, the effect observed in the present study could have been caused by the task in the experiment, which required the participants to react to the color of the bar. Thus, the final bar orientation was more important than the final grasp posture, which may have led to the anticipation of the final bar orientation rather than the final grasp posture. It is possible that what is anticipated (object orientation or grasp posture) depends on the task given. Further studies are needed to investigate whether an initial grasp posture is more likely to be associated with a final grasp posture or a final object orientation.

However, contrary to our expectation, the results show that the reaction times and error rates for prime-target pairs depicting a possible to perform action did not differ significantly from prime-target pairs depicting an impossible to perform action. The fact whether the prime-target pairs represented an action that was possible or impossible to perform therefore had no influence *per se* on the processing of the target images. This finding is not consistent with the results of the studies by Güldenpenning et al. (2012) and Seegelke and Hughes (2015), in which responses to image pairs were faster if they reflected a possible action. While in these experiments, participants had to focus on the movement, in the present study, the aim was to respond to the color of the bar in the target image. In the decision task carried out here, the action or grasp posture was therefore irrelevant for the decision, which may have resulted in a different pattern of results. In addition, no priming paradigm was used in Seegelke and Hughes (2015). The different tasks and paradigms used may have resulted in a different pattern of results.

The reaction times of the implemented control conditions show that only the congruence of the bar color between prime and target had an influence on the reaction times, but not the congruence of the grasp posture. The reaction times are not supported by the error rates, as the control conditions did not differ significantly in terms of error rates. Based on the results of the control conditions, it can be assumed that the perceptual characteristics of the hand had no influence on the response times. Since we did not find any perceptual priming effect of the hand in the control condition, we would argue that the priming effect of the underhand grip within the possible action did not occur due to perceptual processing stages. Additionally, if the priming effect would be due to perceptual priming, we should have found a similar priming effect in the impossible action condition, which we did not. A priming effect of the underhand grip due to a response-congruency effect seems implausible, too, since the top color of the bar in the target image should be responded to and the bar was shown horizontally in the prime image, which is why the prime should not have activated or elicit a response *per se* (i.e., pressing a key on the keyboard). Therefore, we would argue that the priming effect of the underhand grip in the possible action condition did not arise due to perceptual or motor priming but to cognitive (anticipation) processes. These findings support our assumption that the perception of the underhand grip led to the goal-directed activation of the representation of future action states.

The results of the error rates could not fully confirm some significant findings of the reaction times in this study. However, it is important to note that the pattern of the error rates do indicate that the reaction time effects are not based on a speed-accuracy trade-off. A possible reason for the deviation of the error rate results from the reaction times could be the generally low error rates of the study (see also Güldenpenning et al., 2012). The simple task could have been the reason for the few errors. The participants were asked to decide whether the top color of the bar in the target image was black or not. It can be assumed that this decision is relatively simple. In each condition (experimental and control condition), there were at least seven people who made no errors at all. At the same time, 28 of the 30 test subjects showed zero errors for at least one condition. This fact calls into question the meaningfulness of the interpretation of the error rates (Polzien et al., 2019).

To gain further insights into the cognitive processing of grasping actions and to provide conclusions about the underlying mechanisms of the priming effects we found (or the absence of priming effects, e.g., of the overhand grip), future research should also include neurophysiological measurement methods, such as EEG. A further step would be to investigate the relationship between cognitive aspects such as the representation of grasping movements and the anticipatory action planning of grasping. This could be done, for example, with groups of people whose ability of anticipatory action planning during grasping is limited, as is the case for some disorders (Bäckström et al., 2021; Krajenbrink et al., 2021). Another idea for investigating the relationship between the cognitive representation and anticipatory action planning in grasping would be to study people who have a lot of experience with the use of tools (such as craftsmen or blacksmiths). Numerous studies with different levels of expertise in complex sports have shown a connection between cognitive representations and the control and anticipation of movements (e.g., Güldenpenning et al., 2012; Bläsing et al., 2014). Whether so-called experts in grasping movements differ from other groups of people in terms of their action representation and anticipatory action planning of grasping movements has not yet been investigated.

In sum, the results of the present study indicate that the perception of an underhand grip leads to the automatic activation of an action-related representation of grasping actions whose action goal is consistent with the end-state comfort effect. However, it remains unclear whether the anticipated action state is object-related or grasp-related. It is postulated that the initial uncomfortable underhand grip is associated with a final action state that is consistent with the end-state comfort effect and that this anticipative action knowledge (second-order motor planning) is represented in memory. Such a cognitive link between an initial and a final action state might be used to anticipate future action states when selecting a grasp. We therefore assume that the link of different action states in memory might be important for anticipatory action planning during grasping.

## Data availability statement

The raw data supporting the conclusions of this article will be made available by the authors, without undue reservation.

## Ethics statement

The studies involving humans were approved by Ethics Committee of Bielefeld University. The studies were conducted in accordance with the local legislation and institutional requirements. The participants provided their written informed consent to participate in this study. Written informed consent was obtained from the individual(s) for the publication of any potentially identifiable images or data included in this article.

## Author contributions

JK: Conceptualization, Formal analysis, Investigation, Methodology, Project administration, Software, Visualization, Writing – original draft, Writing – review & editing. LV: Conceptualization, Writing – review & editing. TS: Conceptualization, Methodology, Resources, Writing – review & editing.
