# Supplementary material for: Anticipation (second-order motor planning) is stored in memory – processing of grasp postures in a priming paradigm
Source: Front Psychol. 2024 Jul 17;15:1393254. doi: 10.3389/fpsyg.2024.1393254 (PMC11289885; doi:10.3389/fpsyg.2024.1393254)
Supplement: Supplementary file 1 [file Table_1.DOCX]

**Supplementary Table 1**

***Errorrates of the experimental conditions***

| **Possibility** | **Action sequence** | ***M*** | ***SD*** |
| --- | --- | --- | --- |
| Possible action | comfortable-comfortable | 2.75 | 2.01 |
|  | comfortable-uncomfortable | 3.25 | 3.36 |
|  | uncomfortable-comfortable | 2.92 | 3.48 |
|  | uncomfortable-uncomfortable | 3.92 | 4.19 |
| Possible action | comfortable-comfortable | 2.58 | 2.82 |
|  | comfortable-uncomfortable | 3.25 | 3.42 |
|  | uncomfortable-comfortable | 3.92 | 4.24 |
|  | uncomfortable-uncomfortable | 1.75 | 2.09 |

*Annotation. M* = mean value, *SD* = Standard deviation. The values represent the error rates in %.

**Supplementary Table 2**

***Errorrates of the control conditions***

| ***Control conditions*** | ***M*** | ***SD*** |
| --- | --- | --- |
| BC | 1.17 | 2.15 |
| BI | 4.17 | 5.58 |
| BC-GC | 2.50 | 2.63 |
| BC -GI | 1.75 | 2.38 |
| BI-GC | 2.75 | 2.89 |
| BI-GI | 3.50 | 4.98 |

*Annotation. M* = mean value, *SD* = Standard deviation. The values represent the error rates in %.
